# Lectures on Subjects Connected with Clinical Medicine, Comprising Diseases of the Heart

**Published:** 1845-07

**Authors:** 


### ART. XVII.

*Lectures on subjects connected with Clinical Medicine, comprising Diseases of the Heart.* By P. M. LATHAM, M.D. &c. Vol. I.—*London,* 1845. 12mo, pp. 374.

DR. LATHAM, some years since, finding his health impaired by the toils of hospital practice, and feeling laudably unwilling to render his duties as physician easy by indifference to their higher attributes, "relinquished his office at St. Bartholomew's, and with it, some of the best hopes he had of being useful in his generation." The first result of the sacrifice was a return of health,—the next the production of the volume now issued to the world. The author may rest assured that few persons will regard these effects without deep gratification: the gradual restoration to vigour of a distinguished member of the society in which he moves, cannot fail to prove matter for rejoicing among his peers; nor can the appearance of a book pointedly designed and admirably suited to popularise the study of diseases of the heart be regarded otherwise than as a professional event of no slight consequence.

Dr. Latham avows his purpose to be a limited one: it is "to regard the diseases of the heart only in one point of view, i. e. as they appear in the living man. But this one point of view includes the several objects of their clinical diagnosis, and their clinical history, and their medical treatment." And in respect of clinical diagnosis, the writer's faith concerning the means of its accomplishment stands broadly and decisively announced in the words: "enough is already known to make the diagnosis of diseases of the heart hardly anything else than a just appreciation of their auscultatory signs."

One of the characters of this book is the incompleteness of its descriptions;—in no single point or bearing is the subject touched upon fully worked out and evolved. The incompleteness is systematic and designed:

the motives of its adoption we append in the writer's own words. Explanation is indeed here not only interesting and requisite in respect of the book itself, but assumes importance from the fact that the person, who defends and pursues the system in question, once held a prominent place among the clinical teachers of the metropolis.

"But as I lectured, so now I write, for one class of students especially. As my hearers were, so now I presume my readers will be, chiefly those who are seeking information at the bedside. To such there is no greater impediment of knowledge than over-teaching. The teaching which they most require is suggestive. They have the realities themselves to learn from, the original *book* to read, upon which all sound instruction is but a commentary. Therefore the commentator should only interpose when and where he is needed, and not after the manner of certain critics, who most *help* us with their annotations where the sense of the author is clear beyond dispute." (p. ix.)

Now that there is—or rather, according to circumstances, may be—fundamental truth here, we readily admit. But in practice, we apprehend, Dr. Latham's notion would, if carried to its limit, prove subversive of all the good to be derived from the clinical direction of students of disease. In respect of the system of *clinical* teaching prevalent in this country, the idea that its amount is in excess—that youths are misled by being over-led—seems to us not a little paradoxical. That in the Paris hospitals, danger sometimes arises—danger that the mind of the beginner may be confused through the abundance of minute detail, poured forth in almost inexhaustible bounty by enthusiastic and eager aspirants to the renown of first-rate clinical exponents of disease, is—it may without difficulty be conceded to Dr. Latham—an unquestionable fact. But if we turn to the London schools, and mark the style and manner of clinical guidance flourishing *there,*—if we hearken to the occasional lecture, consisting, too often, of a hasty admixture of crudities and common-places, hurried over with an anxiety worthy of a better motive than the always presumable, and too often obvious, one,—if we inquire into the contentment of those taught upon this plan, and find that even they—even those typifications of listless indifference, the "walkers of hospitals"—are dissatisfied, grumble at being *under-taught,* and apply to the editors of weekly prints to urge the claims they have themselves in vain striven to impress upon their so-called teachers,—if all this be done, we affirm the grounds of Dr. Latham's philosophy will, in their practical aspect at the least, appear singularly unstable. So far from agreeing in this matter with this eminent physician, we ourselves have, after no little reflection and observation, persuaded ourselves to regard the very incompleteness, (or, as it is fashionably termed at the present day, *suggestiveness,*) which Dr. Latham would foster, as the origin of our inferiority (where we are inferior) to continental observers. And, on the other hand, our conviction has been deliberately arrived at and is firmly established, that were a system of thorough clinical instruction brought to pervade the schools of this empire, a class of physicians would arise, with the combined scientific and practical attainments of whom foreign nations must hopelessly endeavour—if they did endeavour—to compete. By thorough clinical instruction we mean the conveying of a detailed, systematic, complete history of the particular cases of disease under observation, all the particulars of which history may be and should be (with a few necessary exceptions) ascertained by the student himself.*

But there is another of Dr. Latham's general doctrines of the means whereby practical medicine is to be surely and solidly advanced, that is not only eminently true, but so vitally important, especially at the present period, that we conceive it a duty to aid in its promulgation. We allude to the opinion he holds concerning the degree to which "physiology" should be suffered to constitute the basis of "practical medicine;"—and the opinion is thus pithily and emphatically conveyed:

"It is not *all* physiology which can be made useful towards the knowledge and treatment of diseases, but only those parts of physiology which are undeniably true, and not only true, but *easily and at once seen to be so.* A great deal of what is termed physiology *has turned out to be a mistake;* and so far as it has got mixed up with our notions of disease (and *this has happened to a deplorable extent,*) it has *hindered the progress of practical medicine.*" (p. 9.)

The italics are our own, and we use them to mark our deep conviction of the truth and importance of the words. The lamentable delusion that speculative systems of medicine, based upon the everchanging phantasies of physiology, are calculated to advance the skill of the physician in the management of disease, cannot be too energetically and systematically denounced. Books constructed on this principle are peculiarly dangerous—and dangerous in the direct ratio of their attractiveness from their prettiness, their superficial ingenuity, and their apparent acquaintance with the intimate phenomena of vital action.

We now proceed to give an account of the work before us; and as it is one of no ordinary merit, both as to matter and style, we shall have great pleasure in reviewing it, chapter by chapter, from beginning to end. A volume like the present is a lure somewhat of the rarest for a medical critic to be tempted withal.

Lecture I. Dr. Latham's first lecture embraces the consideration of the natural sounds, impulses, and resonances of the heart, and of the manner in which variations in degree and extent of these are significant of disease.

In the generation of the first sound of the heart in health, muscular contraction, muscular tension, and extension of the auriculo-ventricular valves, are severally engaged; the second sound results from the sudden closure of the sigmoid valves: this is the theory used by Dr. Latham. He conceives the ear must become practically acquainted with these sounds in order to appreciate them justly, and therefore declines giving his pupils or readers any hint concerning their characters. Dr. Latham perfectly corroborates our own experience in respect of the extent to which the first sound is audible in health in the following words: "I am persuaded two healthy persons would not easily be found in whom it would be heard within exactly the same thoracic space." On the limitation of the second sound he attempts greater precision, but the statement that it is "audible

* We cheerfully admit that in certain of the metropolitan schools the system of clinical teaching has undergone marked improvement within the last few years. But much remains yet to be done; not only directly, but indirectly in respect of the over-devotion of students' time to what are termed accessory branches of medical knowledge.

in the *course of the aorta* and of the pulmonary artery and of the carotids" is either meant to be vague, or is liable to erroneous construction.

Lect. II. Sounds of the heart different in kind from its natural and healthy sounds next engage Dr. Latham's attention. All varieties of valvular sound he would designate by the name *endocardial,* all sounds produced in the pericardium *exocardial, murmurs.* We question whether a student is likely to gain information as readily from this mode of styling the unnatural sounds proceeding from the heart, as from the more usual, but, we admit, somewhat cumbersome system. Although the word *exocardial* contrasts very harmoniously with endocardial, in our old-fashioned horror of new words we almost wish Dr. Latham had retained the term *pericardial* for the former. As critics we may also object to the word *murmur* as applied to the pericardial friction-sound and its varieties. As a mere question of words it may and is undoubtedly quite as fair to call a friction sound, as a morbid valvular sound, a murmur; but, in strictness, both should not be designated by the same title, and usage has assigned the one mentioned to the valvular sound.

Dr. Latham gives a sprightly sketch of the progress of the diagnosis of valvular disease, by means of endocardial murmurs, from the first rude essays of Laennec to the existing condition of very notable perfection. The facts are strikingly and instructively put, and the terse language in which they are told, gives them a force that must (presuming them to have been thus orally delivered) have produced its effects on the intelligences of those who listened. But no mention of *names* occurs from first to last. This is a mode of proceeding wherewith we have no sympathy. It is unjust to those laborious men who have toiled to extend the field of our art, —it is impolitic towards those entering on the study of that art, to withhold from the owners the meed of applause which general opinion has awarded.

The infinite rarity of direct or diastolic mitral murmur compared with the frequency of indirect or systolic murmur, connected with the same valve, has struck all observers of cardiac disease. Dr. Latham adopts the following explanation of the fact:

"It is probable that, as in health, when the mitral orifice is entirely free, the blood glides from the auricle into the ventricle without any impelling force from behind; so in disease, when the orifice is narrowed, the resistance does not produce any extraordinary effort on the part of the auricle to overcome it. And thus in disease as well as in health, through a narrow passage as well as a free one, the onward current of blood from auricle to ventricle is still without noise. That it is otherwise with the regurgitating current through the same passage, and that the murmur of the blood rushing back from ventricle to auricle should be often signally loud, must be owing to the force of the ventricle, now engaged in impelling it." (pp. 38 9.)

Having stated the current principles concerning the localization of endocardial murmurs by means of the *time* at which they are heard and the *direction of their propagation,* Dr. Latham proceeds to express his doubts that those principles will invariably lead to correct diagnosis. He is "less peremptory about the certainty of their application than he was a year or two ago." So are we. But still, like Dr. Latham, we abate not a jot of our reverence for the principles in themselves, believing their fundamental truth irrevocably sealed by experience;—we would believe that there are occasional counteracting causes which modify, as a necessary physical result, the direction in which these murmurs are propagated and conveyed. The establishment of the nature of these counteracting causes (rarely influential, as they are, in actual practice) is the existing desideratum. The writer attempts to supply it,—but the attempt is chiefly valuable, as likely to lead others to pursue the subject. Four causes are suggested: the presence of solid substances, exterior to the heart, within the chest, as morbid growths, aneurismal tumours, and condensed portions of the lung; the enlarged capacity of the heart itself; the mere loudness of the endocardial murmur; a peculiar *quality* of the endocardial murmur, giving it a high *musical note.* Quality of sound has, we would observe, nothing to do with its musical note.

Dr. Latham protests against the "popular notion" that endocardial murmurs increase in loudness directly as the amount of disease and consequent impediment. He is right. And he illustrates the objection by reference to two cases terminating fatally at the same period, in one of which there was no endocardial murmur at all, in the other a loud bellows-murmur extensively audible. In the former case, the auriculo-ventricular orifice was so narrowed as only just to admit the little finger, and the aortic orifice was only just not closed; in the latter there remained a tolerably free space for the passage of blood through both orifices. "The truth is," says Dr. Latham, "that the murmur becomes louder as the disease and the impediment increase only *up to a certain point,* and then that it becomes less and less loud as they go on to increase beyond this point."

"There is, or rather, perhaps, there was, a notion that endocardial murmurs have wonderful diagnostic secrets wrapped up in their varieties of *kind* and *quality.*" Whether such notion be past or present, Dr. Latham is perfectly right in maintaining its fallacy. The affectation of assigning the amount and characters of obstruction through the whistling, filing, cooing, &c. varieties of murmur, is among the erroneous puerilities of the auscultatory art.

Lect. III. Dr. Latham proceeds to illustrate the fact that the origin of endocardial murmurs in valvular disease, may be otherwise caused than by valvular impediment, and that their mode of production is in yet other cases doubtful.

There are instances in which endocardial murmur comes and goes; it is not constantly present. It disappears under rest, it appears under exertion. We have known this not *very* uncommonly to be the fact. It is probable in these cases, according to Dr. Latham, that there is a real obstacle "not enough to cause the requisite degree of vibration when the current of the blood is slow and undisturbed, but quite enough when it is more rapid and forcible." This is one explanation,—the phenomena of anæmia have to our minds occasionally suggested another.

An endocardial murmur may arise, Dr. Latham finds, a few days before death independently of cardiac disease,—a fact he is unable to explain.

A loud endocardial murmur may occur in connexion with the hysterical paroxysm,—and mere force of contraction of the organ will produce the same sound in children. We know the facts by experience; we are unable to explain them satisfactorily any more than Dr. Latham.

When the chest is deformed, "there is an end of our pretending how to calculate what its condition may be, by listening and feeling and percussing. Its sounds and impulses and resonances, be they what they may, are now worth nothing at all as guides to diagnosis. The heart is dragged from its proper seat, and imprisoned in some strange place, and perhaps turned almost topsy-turvy by the encroachment of the vertebral column and the approximation of the ribs." In some cases an endocardial murmur may be simulated, if pressure be made, in ausculting, with the end of the stethoscope.

A blowing murmur has been described by some previous writers as seated in the subclavian artery, and attendant in a certain proportion of cases on pulmonary consumption. To this Dr. Latham makes no allusion; but he dwells upon a certain murmur (coincident with the systole of the heart) audible frequently in the same disease in the space comprised between the upper border of the second and lower border of the third costal cartilage an inch to the left of the sternum, as having some diagnostic value. "Supposing the pulmonary artery in its first division to be the seat of the murmur, does it become such in consequence of its own disease, or by reason of pressure or impediment reaching it from diseased lung?"

There is nothing deserving notice in the author's remarks concerning the "possible fallacy" (and we may add the not unfrequently committed error) of mistaking the respiratory murmur for an endocardial murmur. Nor is there anything novel in his comment on anæmic vascular murmurs.

Lect. IV. Here we find Dr. Latham discoursing of the general estimate to be taken of the uses of auscultation applied to the heart. In his wonted lively and dramatic style he traces the advance of knowledge in the diagnosis of chronic cardiac disease, and describes the efforts made to attain the practical benefit of detecting the acute forms of disease in the organ,—those forms that are remediable and curable.

"And at length the mark has been hit, and the prize has been won. For now there is no truth experimentally more certain than this, that auscultatory signs above all others, and oftentimes before all others, and oftentimes in the place of all others, may be safely trusted to declare the beginning and the augment, the decline and the cessation of acute inflammation in those structures of the heart, which are especially, if not solely, obnoxious to it; and that the same signs may be confidently appealed to as guides, by which to choose the remedy, and apportion its quantity and regulate its force, and continue or discontinue its application." (p. 87.)

Lect. V. Dr. Latham in former days believed that the bellows-murmur of cardiac disease accompanying acute rheumatism was seated in the pericardium; subsequently he "began to suspect that it proceeded from the internal lining." But he could not verify his conjecture, for *no patient of his* ever died of that "disease of the heart, which, coming on during acute rheumatism, is characterized by the bellows-murmur." But "what my own experience would not furnish," continues Dr. Latham, "M. Bouillaud's has supplied. Many have died during the active progress of this disease under his care, and dissection has found it to be inflammation of the endocardium. Thus we are indebted to M. Bouillaud for our first knowledge of the important fact." Admitting the inferences inevitably deducible from these statements, it would be a nice question for the casuists to determine which of these two physicians had, in the instances referred to, been the most useful in their spheres.

It will be well for our junior readers to ponder deeply on the following most wise and emphatic words:

"Seeing, then, that the endocardial murmur alone can determine the existence of endocarditis, you are required to search after it in every case of acute rheumatism. I say emphatically *to search after it,* because it is one of those signs which must always be sought before it can be found. It does not intrude itself upon our notice like palpitation, or an irregular pulse. The patient does not draw our attention to it as he does to pain. The physician must make it out entirely for himself. And indeed it is infinitely important that he should have the earliest possible notice of it with a view to the earliest possible application of the remedy. Never omit, therefore, to listen to the præcordial region whenever you visit a case of acute rheumatism, and to visit a case of acute rheumatism oftener perhaps than you otherwise would do merely for the sake of so listening. All may seem to be going on well. The general symptoms may be far from severe. The chest may be free from pain. The heart's action may not awaken suspicion by its force or irregularity. Nevertheless, its internal lining may be inflamed, and, if you listen, the endocardial murmur may convey the momentous fact directly to your ear." pp. 104-5.)

LECT. VI. But not only is endocardial murmur to be sought for, but certain modifications of the natural sound are to be sought for as its inevitable prelude. Dr. Latham hardly ever knew an instance of acute rheumatism in which unnatural length or roughness of sound detected one day, has not become an unequivocal cardiac murmur in twenty-four hours. When he hears such modifications of sound, he consequently begins the treatment of endocarditis at once.

Although endocardial murmur sometimes constitutes the sole evidence of the existence of endocarditis, and though functional disturbance of the organ may be completely absent, this is not the common case. Symptoms are superadded, and these are,—pain and anguish of various degrees and kinds, excessive impulse, and intermittent fluttering action of the heart. These symptoms give us a much surer insight by their amount and intensity into the peril attached to the disease, than does the murmur this evolves. Pain in the precordial region may arise in the course of acute rheumatism without being followed by the auscultatory signs of either endocarditis or pericarditis,—but prudence would lead us to commence the treatment, as if one or other were destined to ensue. We envy the earnestness of conviction which can justify Dr. Latham in using the following language in his comment on the importance of treating such precordial pain, as if significant of already evolved endocarditis: "For thus, if we have begun the treatment *only a single day sooner* than we otherwise should have done, we may have *perfectly cured* the disease, which but for the gain of this single day, would never have been more than half cured." After such an emphatic declaration, the author but weakens his point in adding, "the gain of a single day in the treatment of endocarditis is a gain indeed." The man who believes this, and does not treat his rheumatismal patients at the first onset, must eventually feel bitterly the force of the "diem perdidi" of the Roman.

The consideration of the diversities of relation which the endocardial murmur is found to bear to other symptoms leads Dr. Latham to establish: 1st, that in some cases of endocarditis the murmur is coincident with the very commencement of the inflammation; 2dly, that in some, and those the most frequent, cases it does not arise till the inflammation is somewhat advanced; 3dly, that in some, and those the least frequent, cases it does not arise until the inflammation is on the decline, or has actually ceased; and upon this distinction are founded considerations of interest for which the original should be consulted, but of which we shall give a specimen:

"The endocardial murmur arising under these circumstances was unchanged by medical treatment. It remained as long as the patients continued under observation. The inference from such an event is clearly this, that an inflammation of the endocardium had accompanied the rheumatic fever; that this inflammation was of small activity, and insufficient during its progress to interfere with the natural sensations and movements and sounds of the heart, but enough in the end to produce by its effects some permanent inequality on the surface of a valve, and a permanent murmur as the sign of it." (119-20.)

LECT. VII. We are here brought to the history of pericarditis. Dr. Latham teaches that the similitude of exocardial murmur to the sound produced by rubbing the hands or coat-cuffs together, the sensible nearness of the murmur to the ear applied to the precordial region, its conveyance to a distance over the chest, and its non-conveyance in the course of the aorta and carotids, are sufficient to stamp it as originating in the pericardium. But further, the crumpling, creaking, churning, &c. varieties, less perfect in their character, become changed in the course of one or two days (if arising from pericarditis), completely or partially, into the genuine exocardial murmur. These more indefinite sounds will sometimes come and go for two or three days, and then cease altogether, or then become permanent; such sounds are of pericardial origin.

Dr. Latham illustrates his opinion "that serous effusion within the pleura *always* obliterates the attrition-sound, and that serous effusion within the pericardium *generally* leaves it unaltered," in his usual pointed style. But he must be aware that Dr. Stokes affirms that extensive pleural dulness and friction sound may coexist. If the words *always* and *generally* were transmuted into the phrases *with the rarest exceptions*, on the one hand, and *not unfrequently*, in the other, Dr. Latham's opinion would be more accurately in accordance with fact. The reason, why this should be so, is sufficiently obvious.

Visible undulation and tactile vibration between the cartilages of the left second and third or third and fourth ribs, or between both at the same time, (never occurring in any other situation,) are dwelt upon next. It is to be noticed that Dr. Latham makes no reference to vaulted form of the precordial region, nor to the increased distance at which the heart's sounds appear to be produced from the ear, as among the signs of pericarditis. Yet an observing and diligent investigator of cardiac disease has affirmed of late that such vaulted form is sometimes the very first *physical* peculiarity (including the auscultatory) induced by the disease.

There is no one symptom of inflammation to which ordinary practitioners trust so implicitly, in evidence of the existence and amount of that state, as pain. A more utter error cannot exist; the writings of Louis especially abound with proofs of the error and safeguards against its commission. Dr. Latham thus comments upon the fact in respect of pericarditis:

"But of all symptoms mere pain is the most inconstant and uncertain, whatever be the disease. It is so in pericarditis. It is present in one case, and absent in another strangely and unaccountably. I have known much pain, when the disease has been of little severity, of short duration, and of easy cure; and I have known the severest pericarditis pass through all its stages without pain. All other symptoms have been present to mark its reality and its progress; the murmur and the præcordial dulness, and the fluttering heart, and the respiratory anguish. And sometimes the patient has died, and sometimes he has escaped by a tardy and precarious convalescence. But from first to last there has absolutely been no pain." (pp. 141-2.)

Lect. VIII. Between the years 1836 and 1840, both inclusive, there occurred under Dr. Latham's care at St. Bartholomew's Hospital 136 cases of acute rheumatism. These cases, considered in respect of cardiac affection, are thus arranged by the writer:

| | | |
|---|---|---|
| "Cases of acute rheumatism - - | 136 | |
| Heart, exempt in - - - | | 46 |
| affected in - - - | | 90 |
| Seat of diseases in the heart: | | |
| Endocardium alone in - - | | 63 |
| Pericardium alone in - - | | 7 |
| Endocardium and pericardium in - | | 11 |
| Doubtful in - - - - - | | 9 |

—Deaths 3. In all of whom both endocardium and pericardium were affected." (p. 144.)

From this experience it follows that about two thirds of sufferers from acute rheumatism are attacked with cardiac inflammation. We believe that the proportion is above the usual standard,—a circumstance probably to be explained by the "sedulity of Dr. Latham's clinical clerks who were ever on the alert to gain admission into his wards of (what were esteemed) interesting cases." It further follows from this experience that endocarditis is nine times as frequent in rheumatism as pericarditis; and that pericarditis is more frequently found in combination with endocarditis than alone. This portion of the results does not very materially differ from that of close observers in general.

Of sixty-three individuals with inflamed endocardium not one perished; the disease, even in individuals commonly of deteriorated constitution, (for of such is the population of the London hospitals composed,) is consequently not one involving much peril to life. But the results of another kind are to be considered; restoration of the endocardium to its healthy state was rare. This is seen from the facts, that the membrane remained permanently injured in forty-six cases, and recovered its complete integrity of structure in seventeen only: in other words, endocardial murmur totally disappeared from seventeen hearts only.

In all the seven cases of simple pericarditis recovery took place, and all murmur disappeared. But Dr. Latham is of course unwilling to affirm that all anatomical traces of the disease had, as in instances of cessation of endocardial murmur, disappeared. It is probable that in all, or in very nearly all, such cases some amount of adhesion permanently remains, and that the functional ease and perfection of the heart suffer proportionally.

Of the eleven cases of endo-pericarditis three terminated fatally; in none of the eight instances of recovery does the author believe complete restoration of the structures to the healthy state ensued.

Does ground exist for believing that in cases of these combined inflammations, either naturally tends to produce the other? It appears that in four of the eleven cases the endocardial came on before the exocardial murmur, and in four the latter before the former; while in one they arose simultaneously; and in two they coexisted when the cases were first examined.

Lect. IX. The consideration of inflammation of the lungs accompanying acute rheumatism, either alone, or in combination with endocarditis, or with pericarditis, or with both, furnishes Dr. Latham the opportunity of exhibiting greater originality than in his previous lectures.

Inflammation of the lungs (as Dr. Latham uses the words,) occurred in 24 of 136 cases of rheumatism; in 1 of 5½ cases consequently. The cases were made up of 4 of bronchitis, 18 of pneumonia, and 2 of pleurisy. The probability of inflammation of the lungs arising out of acute rheumatism is small; and the probability of such an event is not at all augmented by alliance of the articular affection with endocarditis. But inflammation of the lungs coexists sufficiently frequently with pericarditis, and with endo-pericarditis, to establish a sort of natural connexion between them. The following table exhibits more strikingly and concisely, than is otherwise possible, these very interesting facts.

"Of rheumatism without affection of the heart there were—Cases 46. Lungs affected 5:—Single pneumonia (fatal;) Single pneumonia; Single pneumonia; Diffused bronchitis ending in double pneumonia; Diffused bronchitis of both lungs.

"Of rheumatism with endocarditis there were—Cases 63. Lungs affected in 7:—Double pneumonia, Double pneumonia; Double pneumonia; Diffused bronchitis passing into double pneumonia; Single pneumonia; Diffused bronchitis of both lungs; Bronchitis passing into inflammation of the larynx and trachæa.

"Of rheumatism with pericarditis there were—Cases 7. Lungs affected in 4:—Double pneumonia; Diffused bronchitis passing into double pneumonia; Single pneumonia: Single pneumonia.

"Of rheumatism with endocarditis and pericarditis combined there were—Cases 11. Lungs affected in 8:—Double pleurisy with double hydrothorax; Single pleurisy with hydrothorax; Double pneumonia; Double pneumonia with double pleurisy and double hydrothorax (fatal); Single pneumonia; Single pneumonia; Pneumonia and diffused bronchitis of one lung (fatal); Diffused bronchitis of both lungs (fatal.)" (pp. 164-5.)

Two interesting cases (one fatal, the other not so,) are next recorded; the details are tolerably close; and the narratives exhibit very remarkably Dr. Latham's confidence in the powers of "the *great* remedy," mercury.

Lect. X. Dr. Latham introduces his account of the treatment of acute rheumatism, (preparatory itself to that of the cardiac inflammations,) by a comment on the fact that venesection, opium, calomel, colchicum and drastic purgatives, have severally worked their cures of the disease,—understanding these remedies to be employed in each instance as the "sheet-anchor" (in the current cant phrase) of the individual prescribing them. Dr. Latham bears his testimony to the truth that under the use of each he has "seen patients *get* well." Has Dr. Latham ever seen patients afflicted with severe acute rheumatism, sent forth from hospital in a state of (to their feelings) complete restoration,—which patients had nevertheless undergone no treatment but that signified by abstinence, the free use of diluents, and the occasional administration of a gentle laxative? Probably he has not. *We* have. And we confess that such sights have shaken our faith,—have shaken it especially when taken in conjunction with the circumstances alluded to by Dr. Latham himself—as to the real part which active medication plays in saving life. Dr. Latham, however, maintains the robustness of his faith unweakened, and in sharp, antithetic diction and earnest and cordial tone, argues the claims of art versus those of nature. We sincerely hope, for their own sakes, that each reader of his pages may share his convictions,—their inward sense of power and satisfaction at the bed-side of rheumatic patients will be infinitely enhanced by such participation. We the more sincerely hope this,—because we have in our own persons often felt the want of this inspiriting faith. Often have we sighed to possess that genius for belief, and that faculty (for it is such) to conceive art all-powerful, that makes the practice of medicine for some men a perpetual triumph.

Yet, even on Dr. Latham's own showing, the treatment of rheumatism on the active, interfering, and nature-guiding principle, is not to be carelessly ventured upon. It is in fact *gros jeu,* this treatment. For we are told "it is often only when the powers of medicine are pressed *even to the verge of destroying life,* that *life is saved.*" If the treatment of rheumatism ever come to this, adds the author, it is right to know what we are about when we undertake to treat it; a truth in which we most heartily coincide.

"Disease is a series of new and extraordinary actions. Each link in the series is essential to the integrity of the whole. Let one link be fairly broken, and this integrity is spoiled, and there is an end of the disease; and then the constitution is left to resume its old and accustomed actions, which are the actions of health." Such is Dr. Latham's version of an old explanation—so-called at least—of the fact that recovery ensues from certain diseases under systems of treatment the most unallied, nay the most opposite in nature. We *could* suggest another and an obvious one,—but we abstain.*

Here is a striking passage, concerning the influence of large bleeding in rheumatism:

"I have seen people enormously bled in acute rheumatism, and their entire disease swept away at once, and health restored rapidly. And the practice which will do this, is it not a splendid and a tempting practice?

"Again, I have seen people enormously bled in acute rheumatism, and their disease swept away at once; but they have forthwith gone raving mad. And a practice which will do this, is it not a hazardous practice?

"And again, I have seen people enormously bled in acute rheumatism, and no single pain has been mitigated; but the disease has continued for an unusually long time in its acute, and then has degenerated into its chronic form. And a practice that has this issue, is it not a doubtful practice?" (p. 193.)

Dr. Latham teaches ingeniously that the evils to be arrested in ordinary inflammation by profuse bleeding, namely, the eventual textural changes

* "Why does bark cure ague, sir?" "Because it interrupts the concatenation of morbid phenomena." Such was the habitual and well-known query (and such the required response) of an Edinburgh examiner for degrees, some years since renowned in that school. Is Dr. Latham's sentence more than a paraphrastic statement of the Scotch elucidation?

produced by the morbid process, are not to be dreaded in rheumatism; and that the motive for pushing venesection to extremes, and until all vascular action have been quelled, does not exist in the latter affection. Employed in moderation, and "rather as preparatory and auxiliary to other remedies, than for its own exclusive power," Dr. Latham objects not to venesection. But he is especially chary of carrying bleeding to such a point as to communicate a shock to the nervous system. He addresses his audience thus: "If you do communicate that shock, you are likely to *disturb the just tenor of the disease,* and then some untoward circumstance may arise." Why was not this very disturbance of this very tenor, set down in the passage just quoted by us (p. 185,) as the means to the end sought by all treatment? We do not thoroughly comprehend this seeming contradiction.

The pith of the comments on the treatment by opium alone is contained in the following words: "I regard the indication found in the nervous system to be upon the whole a safer and better guide for the treatment of rheumatism, than that found in the vascular, and opium upon the whole to be a safer and better remedy than venesection; *if* we are to follow one of the two indications, and to use one of the two remedies only."

The third plan, which attempts the cure of the disease by procuring large evacuations from the liver and intestines, and consists in the administration of ten grains of calomel at night, and a black draught in the morning, repeated as long as they are well borne, is thus eulogized:

"It abates the fever, it softens the pulse, it reduces the swelling, and it lessens the pain. In short, it subdues the vascular system like a bleeding, and pacifies the nervous system like an opiate; and often in the course of a week the acute rheumatism is gone. In three days there is often a signal mitigation of all the symptoms; and in a week I have often seen patients, who have been carried helpless into the hospital, and shrieking at the least jar, or touch, or movement of their limbs, risen from their beds, and walking about the ward quite free from pain." (p. 204.)

But this sort of medication sometimes cannot be borne at all; and if salivation (the chance of which is *always* incurred) arise, the treatment must be changed altogether. In this contingency, therefore, "time is lost, the case is perplexed," [or rather the doctor?] "the disease is *prolonged,* and the patient perhaps *injured.*" Still, concludes Dr. Latham, in calomel and purgatives we have a better mode of treatment than in venesection or in opium. It has appeared to him not only to bring the disease to a conclusion in a shorter time, but to prepare the way for a more rapid convalescence than the other methods.

But, says Dr. Latham, there is a plan of treating acute rheumatism, which is juster and safer, and applicable to more cases and more successful than any one of the three referred to. And that plan is *a compound of all three.*

LECT. XI. The mode in which the three plans are combined by Dr. Latham is gathered from the following passage:

"When from the pulse I have considered venesection necessary to bring down the circulation, the loss of between twelve and sixteen ounces of blood has generally been enough to answer the purpose in view; and the venesection has seldom been repeated.

"The opium, and calomel, and purgatives I have been accustomed to give in combination thus: with the calomel administered at night, according to its quantity, I have united more or less of opium. To ten grains of calomel I have added one grain of opium; or to five of calomel I have added half a grain, continuing to give them together in the same proportions, night after night, as long as they are needed. Then, on each succeeding day, when a large purgation of the bowels has been duly obtained, I have still given the opium alone, or with saline draughts, in doses of half or one third of a grain, every five or six hours. And thus, with the larger quantity at night, and the smaller quantities during the day, about two grains of opium have been commonly taken in the course of twenty-four hours." (p. 214.)

There may have arisen surprise in the minds of some of our readers that colchicum has so far received no mention from the deliverer of these lectures. The delay is by him referred to the circumstance that its curative properties are not, *like theirs,* constantly annexed to any known operation upon particular organs. The reason begs a question, and begs it in our minds in the wrong direction,—and even if the point assumed were correct and granted, the motives for delay were an insufficient and, as a matter of principle, an incorrect one. At least so we believe. And indeed we delight to find that in a subsequent page Dr. Latham admits our implied notion upon the question referred to: "As the disease," he says, "may have an *essential element* beyond its sensible actions and sufferings, so the remedies may have secret operations beyond those which are seen and palpable. And it may be in virtue of those that they cure, and not of these." (p. 227.) The wonder to us is that any human being who has seen—seen with his understanding—twenty cases of disease in his life, can doubt that that *essential element* exists. And the further wonder to us is that men of high intelligence—men of the stamp of this author—will persist in attempting to explain what in the present state of elementary knowledge is utterly inexplicable—the action of alleged remedial agents. Such explanations do very well for the class-rooms of teachers of materia medica; but they savour too much of essential platitude to be suffered to wander beyond those learned precincts.

Colchicum cannot, the writer thinks, be safely trusted single handed for the cure of rheumatism in the severer cases, but it can in the milder ones. In the former class of cases he reserves it for special emergencies, and then employs it with almost unlimited trust and confidence. He "invokes the aid" of colchicum, (uncombined with either alkali or opium,) when fever, pain, and swelling, though abated, have not altogether disappeared, or when pain and swelling do not subside in proportion to the diminution of vascular action. In cases of relapse he has likewise recourse to colchicum. He appears to make no distinction in respect of curative influence between the wine of the seeds and of the root.

Dr. Latham guards his auditors against the notion that all cases of acute rheumatism are as readily amenable to treatment, as his previous declarations on the matter might lead inexperienced persons to suppose. He enters into most valuable considerations on the uncertainties of the art of treatment,—considerations which are often strikingly contradictory in spirit (Dr. Latham is too good a logician to allow them to be so in letter,) to the confident announcements that precede, and that we have glanced at, as we passed. We cannot refrain from transcribing a passage that conveys the essence of several very instructive pages.

"There is no such thing as calculating the results of medical treatment with certainty. Success and failure run contrary to expectation sometimes in every disease, but most of all in acute rheumatism. Where you would look for failure, you often meet with success, and vice versâ. Of rheumatism it may be said generally, that it is less within reach of the remedy in proportion as it is seen and treated at a period more distant from its commencement. Yet loss of time does not augment the difficulties of after-treatment, or diminish the probabilities of its success to the same degree in acute rheumatism as in other diseases of an inflammatory nature. I have often known acute rheumatism of the severest kind have the start of the remedy full ten days or a fortnight, during which nothing whatever has been done for its relief; and, when at length the remedy has been applied, it has been cured as easily and rapidly as I could promise myself that it would have been, had I taken it in hand ten days or a fortnight sooner." (p. 223.)

Infinitely true all this! But how comes it then that the same teacher who tells these important truths, can insist for hours on the certainty of his art, speak of "dealing (by medication,) with blood-vessels and with nerves, and with secreting organs separately," as though all these were so many dead tissues to be manipulated on a dissecting-table? Whence draws he the courage to assure youths that their "different remedies, once set a-foot and pursuing different paths, meet and end in one purpose—and that purpose the cure:

'As many arrows, loosed several ways,
Fly to one mark.'"

Ah! Dr. Latham, you deal here with matters of imagination, and naturally appeal to the poets for illustration.

Turning to the treatment of the cardiac inflammations, Dr. Latham takes occasion to object to the prevalent mode of speaking of endocarditis and pericarditis as "*incidental*" to acute rheumatism. "Who shall say," he inquires, "that endocarditis and pericarditis are not equally *essential* to it with inflammation of the joints?" what he means by suggesting that "both are derived from the attendant fever," is to us incomprehensible, but in this notion that in all probability the primary essence of the malady lies in the blood, there is abundant motive for coinciding.

Lect. XII. Whenever endocarditis or pericarditis form a part of acute rheumatism *practically*, they generally are added during the progress of the latter to the general disease. Sometimes however, the articular and cardiac affections supervene simultaneously, and in some still rarer cases, the cardiac take the lead. Such is Dr. Latham's experience.

The poor are the most frequent subjects of acute rheumatism, and "the time of the heart's immunity" has already, in upwards of half the cases, passed over when they are admitted into hospital. In other words, the physical signs or the functional disturbances of cardiac affection have already appeared, when the physician obtains the opportunity of acting on the general disease.

But in instances which come before us, wherein cardiac affection has certainly not yet supervened, are there any means whereby it may be warded off? Dr. Latham most legitimately rejects the vague statements that have been promulgated concerning the efficacy in this direction of large doses of opium. And on the other hand, he believes the accusation against bloodletting, as favouring the extension of the disease from the joints to the heart, perfectly unfounded. Whatever be upon the whole the best treatment of acute rheumatism, he maintains (and we fully coincide with him) may be considered the best safeguard against its extraordinary perils.

But if the disease have actually announced itself in the cardiac membranes, what is to be done? A preliminary question is, what is to be considered to announce the disease? First, functional disturbances of the heart, though so lately unattended with physical sign; secondly, prolongation and harshness of the systolic sound; thirdly, an indefinable but unnatural sound heard in some limited spot of the precordial region; or fourthly, and *à fortiori*, an endocardial or exocardial murmur.

"When the question is of the joints, it might be laid down as a maxim of practice, to treat the rheumatism, (or the general disease,) and let the joints take care of themselves. But when the question is of the heart, the maxim might be stated conversely; to treat the heart and let the rheumatism take care of itself." So says the lecturer; and he says too that bloodletting, mercury, and opium are the remedies for cardiac inflammation as for rheumatism;—but the remedies are to be differently employed.

As soon as inflammation is known or suspected to have reached the heart, mercury must, according to Dr. Latham, be given without delay for the purpose of producing salivation. And salivation may ensue in "a day or two, or not until after several days, or a week, or after several weeks, or it may not arrive at all." Of a surety it does, as Dr. Latham "confesses," require thorough confidence in this remedy to give it for weeks, waiting, waiting, waiting "for its ultimate effect, when in the meantime it displays no proximate or intermediate effect as an earnest of its curative operation."

Venesection must be repeated again and again, "until the hardness and fulness of the pulse are much abated,"—because the disease to be combated is an inflammation. But the main object in bleeding appears—with Dr. Latham—to be to aid mercury in producing its special action.

"In men of florid aspect and full blood-vessels, though *bleeding* has not been needed for its own sake, yet has it oftentimes been *moderately* used for expediting the sensible effects of mercury. And the sensible effects thus induced have been at the same time curative. But, if the body be first made exsanguine by the lancet, you may gain the *sensible* effects of mercury, and lose the curative; for the two do not of necessity go together." (p. 250.)

Abstraction of blood by cupping or leeches or both may be needed as auxiliary to venesection; or these means may be in themselves sufficient in the way of removing blood. When the general reaction is controlled, but the local symptoms abide; or when the inflammation does not decidedly react on the system at large, then local bleeding may be had recourse to. And on the choice of leeching or cupping it is said: "when the pain or anguish, or by whatever name you call the distress, immediately referrible to the heart, begins suddenly, is at once felt severely, and augments rapidly, then cupping is the remedy; but when it comes on by little and little, and increases slowly, and has not yet reached a great amount, then leeches are the remedy. But leeches are often needed as auxiliary to cupping, just as cupping is to venesection." When Dr. Latham directs cupping in inflammation of the cardiac membranes, he has the glasses applied between the left scapula and spine: the motive and reason are obvious.

Opium is given to calm the nervous system, to abate pain and aid the effect of mercury, and in a full dose is the best means of relieving that sudden anguish which sometimes seizes the heart, and is nothing more than an attack of angina pectoris, superadded to the cardiac inflammation.

It is pointedly shown in this lecture how and why the *symptoms* are more amenable to these treatments than the *signs;* the former are vital, the latter mechanical.

Lect. XIII. The lecturer digresses into an investigation of the general question of mercury as a remedy for inflammation. By many this lecture will be highly prized. In our minds it is less novel, less striking, less logical, and less convincing than any of its fellows. Dr. Latham attempts to reconcile discordant views on the remedial influence of this agent, but adduces no fact which is not familiar to those who hold the said discordant views. That inflammations which tend to the exudation of coagulable lymph, that inflammations localized in serous membranes, that inflammations occurring in individuals of sound and robust constitution, are more readily brought under the influence of mercury than those of the converse or of other descriptions, is assuredly (whether it be true, or whether it be false) no novel doctrine. Nor has it been in anywise, or in any degree through ignorance that these divarications of inflammations, in respect of their submission to mercury, had been suggested as a fact capable of demonstration, that some men refuse to credit the alleged prowess of that mineral. *All* are aware of the suggestions; *some* receive, *others* reject them. Dr. Latham's chapter will not (if we judge aright) lessen the number of the latter by a single integer.

Again, Dr. Latham believes that mercury has two ways by which it contributes to the cure of inflammation. In the one it "constrains the morbid energy of the blood-vessels" (antiphlogistic way;) in the other it aids the reparation of parts by promoting the removal of substances foreign to them (reparatory way.) But so far from being new, this is matter of scholastic instruction. Youths are nurtured in the creed; whether they cleave to it in manhood and old age is another question. And even if they did, their steadiness might be regarded rather as the result of prejudice and habit than of conviction and experience. It is indeed more than conceivable that individuals instructed by one whose convictions are so curiously strong and so emphatically expressed as Dr. Latham's, shall, unless their own minds be of a not very usual stamp, cling to what they *were told,* and reject what they *see.* But in point of fact, what is the worth of *conviction*—be it the conviction of whom it may—concerning the agency of any medicine? Is the time so very long passed, when the *conviction* of the most eminent curers of chancre was, that lavish administration of this same mineral mercury was the only means of curing the primary disease and of warding off those secondary effects which constituted the truest and most baneful horrors of infection? And yet now, at the hour we write, it is known, it is felt, and it is irrevocably established, that there are other agents capable of arresting the progress of the local and primary malady, and that, so far from mercury charming away the risks of diseased bones and other miseries, it, the "great remedy," the "venereal panacea," is their very, their most effectual cause. And to what is due the detection and exposure of these errors? To the deliberate rejection of *convictions* and *authority* as a first step; and to the application of a careful system of comparative *observation* as a second.

LECT. XIV. But Iritis? "If any of you have imbibed an unlucky scepticism," says the lecturer, "respecting the curtaive powers which belong to mercury, a month's diligent attendance at the Eye Infirmary will be sure to disabuse you of it." We deny the justness of the prediction. And we deny it on grounds taken by Dr. Latham himself in his pleadings for the mineral. He tells us the seat of the inflammation is all important. He makes the most notable distinction between inflammations affecting serous and mucous membranes. And why may we not push his own principle further? We do so: and we tell him in turn, that though he prove to us that mercury possesses magical powers in controlling iritis, he gains no whit in our convictions as to its efficacy in curing inflammations in general. Nor will he so gain, unless in the instance of persons ready to accept *analogy* as *identity*, and *possibility* as *demonstration*. Let it be understood that we pretend not in the remotest degree to contest the statements made by surgeons concerning iritis and its cure by mercury; but we say, the alleged effects in iritis have been *observed*,—they rest not for credence on *analogy* or *ratiocination*.

LECT. XV. Dr. Latham finds in his clinical records series of cases of rheumatic endocarditis referrible to the following heads: (*a*) Cases in which bleeding and common antiphlogistic remedies alone were employed without a grain of mercury being given, and in which all requisite evidence of perfect cure ensued. But he finds no cases in which mercury alone was given, without any antiphlogistic measures being had recourse to, and in which frequent reparation followed. While then he possesses "facts which claim an independent remedial power for bloodletting, he has none which claim the same for mercury." (*b*) Cases in which bleeding and mercury were employed conjointly, and the evidence of cure was satisfactory; the mercury here had produced no salivation. Here, in his own words, "one cannot be sure that the mercury had any share at all in producing the result." (*c*) Cases in which bleeding and mercury were employed conjointly and salivation quickly followed, and every vestige of the disease was swept away at once. But "no opinion could be formed how much of the cure was due to the bleeding, and how much, if any part at all, was due to the mercury."

"Again, I find some cases in which bleeding and mercury were employed conjointly, and salivation followed, but it was slow to arrive. And reparation was complete in the end, but it was after a long time. Here the manner and gradations by which the disease declined appeared to correspond with the sensible operations of the remedies, and to denote, with seeming exactness, the curative influence belonging to each. The bleeding was practised, whereupon vascular action immediately abated much of its force, and pain, and palpitation, and dyspnœa, immediately went away, but the endocardial murmur remained. Mercury, too, was given from the first, and day after day it was still given, yet there was no salivation. At length, however, salivation arose, whereupon the endocardial murmur ceased." (p. 298.)

Now in the remarks made on these series of cases Dr. Latham abates some slight amount of the pretensions set up for mercury, in the following wise:

"If I were called upon to bring sure proof of the remedial power of mercury in endocarditis, the last (*d*) perhaps, are the only cases to which I should be allowed to appeal; and these claim for it (what I have explained to be) a reparatory, not an antiphlogistic power. They do not satisfy us that it had anything to do in counteracting the progress of the inflammation. They only show us that it came in aid of nature in restoring the endocardium to its integrity, after the inflammation had ceased." (p. 299.)

But further on, language less peremptory still is held; the "*reparatory* power of mercury in endocarditis" is said to be "tolerably certain." And finally the author admits (p. 300) that he cannot from his own experience "pretend to have found a certain proof that mercury is an indispensable remedy to the cure of endocarditis." Yet, notwithstanding this, he "still fears to omit its employment in any case of endocarditis with which he has to do." This appears strange; the why and wherefore of the apprehension is left us to divine.

But Dr. Latham turns to a mode of argumentation in favour of the use of mercury of a totally different stamp—the only true mode of settling questions of therapeutics. He counts cases; roughly it is true, but still he counts, and compares. He says, on the one hand, that he has treated many cases of endocarditis with mercury, and that in not one instance has the disease proved fatal; whereas, on the other, M. Bouillaud, who employs copious and repeated bleedings with all the antiphlogistic adjuncts, but without a particle of mercury, has lost several patients. Now here are two series of comparable facts, and they are worth volumes of argumentation. They settle, beyond the chance of a cavil, this point—that the treatment of Dr. Latham is vastly superior to that of M. Bouillaud. But what is M. Bouillaud's treatment?—in plain English bleeding his patients to death. If an individual seized with endocarditis have every drop of blood in his body withdrawn for its *cure*, shall we ascribe his *death* to endocarditis or hemorrhage? Scarcely need we alter the terms of the hypothesis to make it strictly and literally applicable to M. Bouillaud's treatment. Life ebbs *à vue d'œil* under the unrelenting lancet of the member for Angoulême; and we have looked on in bewildered wonderment at the pertinacious adhesion to a practice that, on pretence of curing, slays. Consequently, in proving, as he most unquestionably does, that mercury, opium, and moderate bleeding are used with results more satisfactory than the bleedings of M. Bouillaud, Dr. Latham does little towards elevating a pedestal to the god mercury.

In pericarditis (eighteen cases, complicated and uncomplicated) mercury was always employed, but conjointly with other remedies; Dr. Latham's experience consequently furnishes no evidence of the curative effects neither of mercury or of other agents, taken separately. It would be putting the claims of mercury in an unfair light, if we did not state that in two of the three fatal cases of pericarditis treated by Dr. Latham, mercury, though elaborately poured in, did not succeed in producing salivation.

Dr. Latham's "experience (as far as it goes) tells him, that whenever the exocardial murmur has ceased, early salivation has taken place." But early salivation does not (would that it did!) involve early cessation of the exocardial murmur. His records supply the following basis for these statements:

| | | | | | |
|---|---|---|---|---|---|
| "Case. | 1st. | Salivation produced in | 1 day. | Murmur ceased in | 4 days. |
| " | 2d. | " | 2 days. | " | 7 |
| " | 3d. | " | 3 days. | " | 4 |
| " | 4th. | " | 4 days. | " | 28 |
| " | 5th. | " | 5 days. | " | 14 |
| " | 6th. | " | 5 days. | " | 25" (p. 308.) |

Such were the results when mercury produced salivation *rapidly*. But in five cases it did so *slowly;* that is, it took in one case eight, in two eleven, and in two thirteen days to affect the gums. But Dr. Latham assigns the mineral nevertheless an important influence here; in doing so he places himself somewhat in the position of defending propositions not actually contradictory, but still not easily reconcileable. For we are first told that the occurrence of salivation is the mark of curative influence, and the mineral is excused in two cases (referred to just now) for not curing, because it could not succeed in salivating; and then we learn that it finishes off cures even where it is excessively slow to salivate. "Mercury in the five cases *took up the cure*, where common antiphlogistic remedies had left it, and came in with its peculiar power and efficacy to complete what they were not able to accomplish. And *then* inflammation ceased and respiration began."

In fine the lecturer compares the results of non-mercurial and mercurial treatment thus:

"In foreign practice, no mercury is used from first to last, but all the power of common antiphlogistic remedies is brought to bear upon the disease; and thus its symptoms are mitigated or subdued; yet they return again and again, and are again and again mitigated or subdued. And so the patients are kept alive for a week or ten days, and then they die in the great majority of cases.

"In English practice, mercury is given from first to last. But it is for a long time as if it were not given at all, for it produces no sensible effect. Common antiphlogistic remedies, however, are able again and again to mitigate and subdue symptoms; and so at the end of a week or ten days the patients are still alive. Yet they are ready to die; but in the great majority of cases they do not die. Salivation arrives late, and seems to save them." (p. 320.)

But while we take to ourselves merit for the cure of pericarditis, we should not forget the extent to which pericarditis is capable of existing, and, independently of treatment of any sort or kind, leaving the life of the individual it attacks immediately unscathed. Among 1263 individuals, whose cases were collected from various sources by M. Louis,* and in which post-mortem details were given, seventy had been affected with pericarditis at some period of their existence, for the pericardium presented adhesions in seventy of the whole number. And here, be it observed, M. Louis pointedly says he makes no mention of the minor results of inflammation (the white patch.) Now these cases were observed and recorded at a period when the disease was never treated expressly, for the simple reason that it was never discovered. M. Louis concludes from elaborate examination of the facts in various points of view, that pericarditis left to itself does not terminate fatally in more than one sixth of the cases.

If Dr. Latham's statement that in England the "*great majority*" of cases of pericarditis terminate favorably, be correct, what a terribly frequent disease this must be! For observe the mortality ascribed to it in

* De la Péricardite, in Mém. Anat. Pathol. p. 289; Paris 1826.

the Mortuary Registers for four years; we place rheumatism of course along with it, for it is the pericarditis or endo-pericarditis that kills and not the rheumatism *per se*.

*Registered Deaths from*

| | Pericarditis. | Rheumatism. | Totals. |
|---|---|---|---|
| In 1837 | 103 | 874 | 978 |
| 1838 | 124 | 1030 | 1164 |
| 1839 | 135 | 946 | 1081 |
| 1840 | 165 | 962 | 1127 |

These numbers are for reasons, more or less obvious to every one, and well known to those in the habit of consulting the Registers, certain to be somewhat, and likely to be considerably, below the real mark of frequency.

LECTURES XVI and XVII. The last two lectures are devoted to endocarditis and pericarditis occurring independently of rheumatism. We must leave these unnoticed, not from any deficiency of interest in them; but because we have already given the work all the space we can afford.

Our task in reviewing this book has been one of unusual gratification. Whether in assenting or dissenting, praising or blaming, we could never for a moment cease to feel that we were dealing with a work, massive and genuine in its substance, chaste and elegant in its form,—a work wrought by a man of talent, an accomplished and finished scholar. The style is in the highest degree correct and polished, richly vernacular and idiomatic, yet imbued throughout with the undefinable charm of antique classicality. At the same time it must be admitted, that delightful as it is to read, the language of this book is peculiar and savours somewhat of mannerism. Dr. Latham delights in antithesis and figure, in pithy apophthegms, in studied repetitions. To some readers, the perpetual antithesis might be fatiguing, and the frequent quaintnesses have the air of affectation; but we pronounce the book to be one of which our medical literature may be justly proud, and we rejoice to be able to class its author among British physicians. Did the profession possess within its ranks even a reasonable proportion of members of the high mental culture of this writer, its position in public estimation would be very different from that it now holds. It is only reviewers and critics—men compelled by their vocation to read (or to try to read) all that issues from the medical press,—who can truly know the full extent of the deficiencies of the profession as to mental cultivation. They, in the course of even a few months, see enough to convince them, that however desirable, just, and necessary, Medical Reform—taken in its ordinary meaning,—may be,—there are other things more necessary still, to emancipate and elevate and dignify the profession;—and these things are to be found only in a GOOD PRELIMINARY EDUCATION. It is vain to bestow on us titles and rank, to enrol us in colleges, to give us representative rights and corporate equality, to throw around us the protection of penal statutes, and to compel the public to yield us our financial dues,—if our education leaves us below the level of the literate classes of our time. If the feelings, tastes, and intellectual faculties are imperfectly cultivated, if the reasoning powers are undisciplined, if men neither know how to observe facts, nor to deduce inferences from them when observed, if they prove by the books which they write, that they know neither their own language nor any other—have they any right to expect, that the world should spontaneously accord to them that respect, honour, and consideration, which the Polished, the Learned and the Wise, compel the world to yield to them?

As appears, indeed, from what has been stated concerning the author's own estimate of his labours, Dr. Latham's work may be looked on as a *cardiac pathology made easy.* But it is much more than this. It is a deeply reflective volume, replete with results indicative of close observation, and abounding with hints and directions which cannot fail to give a strong and beneficial impulse to the study of cardiac disease.

---

